# Transcriptional regulation of *PRPF31* gene expression by MSR1 repeat elements causes incomplete penetrance in retinitis pigmentosa

**DOI:** 10.1038/srep19450

**Published:** 2016-01-19

**Authors:** Anna M. Rose, Amna Z. Shah, Giulia Venturini, Abhay Krishna, Aravinda Chakravarti, Carlo Rivolta, Shomi S. Bhattacharya

**Affiliations:** 1 UCL Institute of Ophthalmology, University College London, London, EC1V 9EL, UK; 2 Department of Medical Genetics, University of Lausanne, 1005 Lausanne, Switzerland; 3 Department of Cell Therapy and Regenerative Medicine, CABIMER, 41092 Seville, Spain; 4 Johns Hopkins University School of Medicine, Institute of Genetic Medicine, 733 N. Broadway MRB 579 Baltimore, MD 21287, USA

## Abstract

*PRPF31*-associated retinitis pigmentosa presents a fascinating enigma: some mutation carriers are blind, while others are asymptomatic. We identify the major molecular cause of this incomplete penetrance through three cardinal features: (1) there is population variation in the number (3 or 4) of a minisatellite repeat element (MSR1) adjacent to the *PRPF31* core promoter; (2) *in vitro*, 3-copies of the MSR1 element can repress gene transcription by 50 to 115-fold; (3) the higher-expressing 4-copy allele is not observed among symptomatic *PRPF31* mutation carriers and correlates with the rate of asymptomatic carriers in different populations. Thus, a linked transcriptional modifier decreases *PRPF31* gene expression that leads to haploinsufficiency. This result, taken with other identified risk alleles, allows precise genetic counseling for the first time. We also demonstrate that across the human genome, the presence of MSR1 repeats in the promoters or first introns of genes is associated with greater population variability in gene expression indicating that copy number variation of MSR1s is a generic controller of gene expression and promises to provide new insights into our understanding of gene expression regulation.

Incomplete penetrance is an almost universal feature of monogenic disorders, yet the molecular basis underlying this phenomenon remains obscure in all but a few cases. Beyond the scientific importance of understanding how gene mutations can be exacerbated or rescued, its clinical importance should not be underestimated. Even for monogenic disorder, the lack of prediction of disease penetrance and severity frustrates genetic counseling. A sentinel example of incomplete penetrance is *PRPF31*-associated retinitis pigmentosa, where presence of symptomatic and asymptomatic mutation carriers is a universal feature in affected families. It has been proposed that phenotypic discordance does not only arise as a result of coding mutations with differential loss-of-function, but also from variation in the regulatory sequences of the same gene^1^. In this work, we provide proof of this theory.

Retinitis pigmentosa is a genetically heterogenous group of retinal degenerations, characterized by progressive cell death of the rod, and then cone, photoreceptors. This leads to progressive nyctalopia, constriction of visual fields and debilitating visual impairment. Autosomal dominant retinitis pigmentosa (adRP) accounts for over one third of cases and mutations in at least 25 genes have been implicated. Intriguingly, mutations in six ubiquitously expressed splicing factors are responsible for adRP (*PRPF3*, *PRPF6*, *PRPF8*, *PRPF31*, *snRNP200* and *RP9*). Why mutations in such “housekeeping” genes produce a retina-specific phenotype is currently unknown. Mutations in *PRPF31* underlie a major adRP locus, termed RP11 (chromosome 19q13.4, OMIM#60138)^2^,^3^. *PRPF31* protein interacts with the U4/U6.U5 tri-snRNP, the central ribonucleoprotein complex of the spliceosomal machinery. *PRPF31* contains a domain that binds the U4 snRNP within the U4/U6 di-snRNP and bridges the U4/U6 di-snRNP with the U5 snRNP to form the U4/U6.U5 tri-snRNP^4^,^5^,^6^,^7^.

Mutations in *PRPF31* are the second most common cause of adRP, accounting for up to 10% of cases^8^,^9^,^10^,^11^. A diversity of mutations has been described, including nonsense, missense, frameshift and large deletions. The most intriguing feature of *PRPF31*-associated adRP is incomplete penetrance, whereby even within families, some mutation carriers are blind whereas others are fully sighted and asymptomatic. Previous work has shown that the asymptomatic trait is strongly linked to inheritance of the wildtype *PRPF31* allele from the unaffected parent^12^,^13^. Haplotype analysis indicates that symptomatic and asymptomatic siblings consistently inherited different wildtype 19q13.4*/PRPF31* parental alleles, suggesting the existence of an inherited parental factor that can “rescue” the disease phenotype^12^,^13^. The expression of the wildtype *PRPF31* allele is more than two-fold higher in asymptomatic individuals as compared to their symptomatic relatives across families^14^,^15^. We hypothesized, therefore, that the major rescue factor (allele) must be common and act in *cis* to increase *PRPF31* transcription. Identifying this element is a priority since it provides insight into “natural gene therapy.” Like all genes, a continuous distribution of *PRPF31* expression is observed in the population and so *PRPF31* transcription must be controlled by multiple genetic elements^16^. Nevertheless, for analyses, we focused on the core promoter region of *PRPF31*, termed BiP (−397 to +539 bp of the annotated transcription start site, TSS, which controls basal gene expression) and a cluster of MSR1 elements located ~630 bp upstream to the TSS (~200 bp upstream to the start of the core promoter region) that exhibits copy number variation (CNV)^17^.

We now show that: (1) the human population harbors 3 or 4 copies of the MSR1 element with the 4 repeat allele conferring increased *PRPF31* expression; (2) the 3 repeat allele quenches gene expression *in vitro*; (3) symptomatic individuals do not carry the higher-expressing 4 repeat allele; and (4) the frequency of asymptomatic individuals in different populations are correlated with the frequency of the 4 repeat allele. This study demonstrates how variation in a minisatellite repeat, widely distributed across the genome, is a major determinant of disease penetrance and suggests that it is a candidate for similar phenomenon at other loci.

## Results

### MSR1 element CNV controls level of gene expression

**Figure 1:**
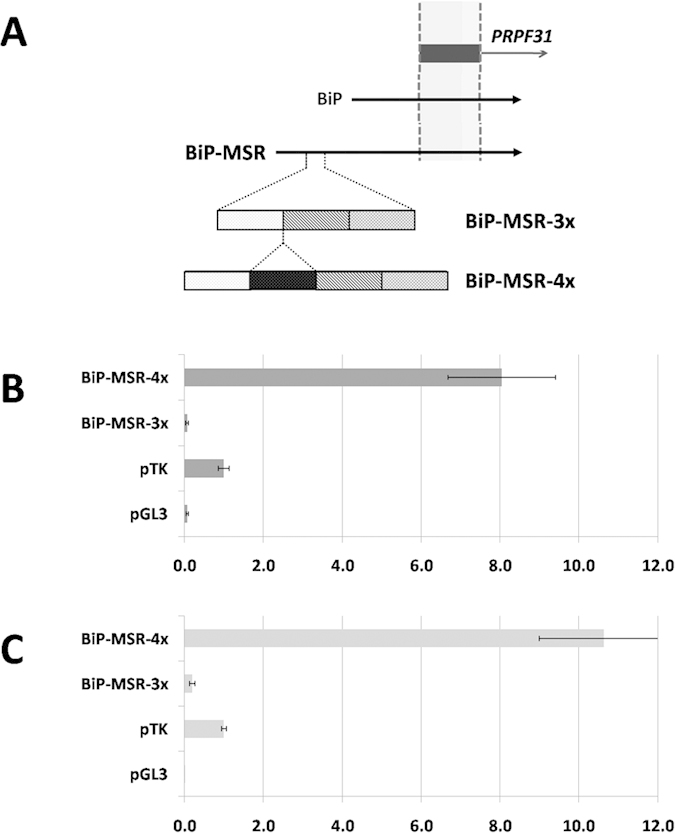
MSR1 element copy number variation was demonstrated to be the major factor controlling incomplete penetrance in RP11. (**A**) Schematic representation of BiP-MSR. The location of *PRPF31* exon 1 is shown, alongside BiP (the previously defined core promoter fragment^15^) and the relative location of the MSR1 elements. (**B**) Dual-luciferase reporter assay of BiP-MSR in RPE-1 cells, presented as the mean ratio of the vector construct to pTK, a basic promoter vector, together with one standard deviation. The positive (pTK) and negative (pGL3) controls are shown; (x-axis – fold-induction over pTK; y-axis – vector). (**C**) Dual-luciferase reporter assay of BiP-MSR in HeLa cells, presented as the mean ratio of the vector construct to pTK, a basic promoter vector, together with one standard deviation. The positive (pTK) and negative (pGL3) controls are shown; (x-axis – fold-induction over pTK; y-axis – vector).

A father-daughter pair were selected for further molecular analysis, as these individuals harboured a 112 kb deletion at the chromosome 19q13 site and so are hemizygous for *PRPF31* and the upstream region of interest^18^. The symptomatic daughter was hemizygous for 3-copies of MSR1 while the asymptomatic father was hemizygous for 4-copies of MSR1. This suggested that the MSR1 CNV might alter gene expression and be the basis for the observed phenotypic difference. We designed the fragment BiP-MSR, encompassing the MSR1 cluster and the full experimentally defined *PRPF31* promoter (Fig. 1A), which was amplified using DNA from the symptomatic daughter harbouring the reference sequence (3x MSR1; BiP-MSR-3x) and her asymptomatic father (4x MSR1; BiP-MSR-4x).

The *PRPF31* core promoter, previously defined as BiP, had ~8-fold induction over the pTK control^17^. The MSR1-containing fragments of interest were cloned into the pGL3 vector and dual-luciferase reporter assays were performed in HeLa and RPE-1 cell lines. In both cell lines, BiP-MSR-4x had strong reporter activity [10.63 ± 1.63 (HeLa); 8.05 ± 1.36 (RPE-1)], and was similar to the BiP promoter (Fig. 1B,C, Table 1)^17^. Strikingly, BiP-MSR-3x had no luciferase reporter activity *in vitro* [0.20 ± 0.07 (HeLa); 0.07 ± 0.03 (RPE-1)] (Fig. 1B,C, Table 1). In the HeLa cell line, BiP-MSR-3x had 53 times lower reporter activity than BiP-MSR-4x, a highly significant difference (Mann-Whitney U = 168, n1 = 14, n2 = 12, p < 2 × 10^−7^). An even greater difference in activity was observed in the RPE-1 cell line, where BiP-MSR-3x had 115 times lower activity than BiP-MSR-4x (Mann-Whitney U = 306, n1 = 18, n2 = 17, p < 4 × 10^−10^). Thus, *in vitro* and in the natural relation to the *PRPF31* core promoter, decreased copy number of MSR1 elements is associated with almost complete abrogation of *PRPF31* transcription.

### MSR1 copy number is a major genetic determinant of the *PRPF31*-associated adRP phenotype

In the general population, in individuals of European ancestry, the minor allele frequency (MAF) of the 4 MSR1 allele is 0.154 with substantial numbers of heterozygous (22.2% are 3/4) and homozygous (4.2% are 4/4) individuals. Consequently, we could search for associations between this common allele and disease penetrance with high statistical power. We studied 42 symptomatic and 29 asymptomatic individuals of European ancestry carrying a *PRPF31* mutation and discovered that all symptomatic individuals had the 3/3 genotype, a highly significant increase as compared to ethnically-matched controls (z = 3.84; p = 0.0001). In contrast, among asymptomatic *PRPF31* mutation bearers, the 4-copy allele was present in the heterozygous or hemizygous state in 8/29 individuals (27.6%; 7 individuals were heterozygous, 1 individual was hemizygous), the remaining individuals being 3/3: the proportion of heterozygotes was significantly higher as compared to ethnically matched controls (z = 2.31; p = 0.02). The pedigrees of asymptomatic individuals carrying the 4-copy allele confirmed that the 4-copy allele was carried on wildtype chromosomes in all cases.

We evaluated a diagnostic test model where a positive result (symptomatic) was having only 3-copy alleles (homozygous or hemizygous) and a negative result (asymptomatic) was having at least one 4-copy allele (homozygous, heterozygous or hemizygous). Our data estimated the positive predictive value at 67% (95% CI = 53.7–78.0%) and, most usefully, the negative predictive value at 100% (95% CI = 63.0–100%). This diagnostic test would be improved by analysis of a larger number of *PRPF31* mutation carrying families. Furthermore, a diagnostic test that uses MSR1 genotype, together with other identified risk alleles, will greatly improve genetic counseling to families.

### Disease penetrance correlates with population frequencies of MSR1 alleles

To further investigate the role of MSR1 in the incomplete penetrance of *PRPF31*-associated adRP, we examined the pedigrees of all families of European and Asian (Japanese/Chinese) origin available to us for asymptomatic mutation carriers. A total of 28 families of European origin with 270 mutation carriers were analysed, of which 92 (34.1%) were asymptomatic (Supplementary Data 1; Figure S1). Twelve families of Asian origin have been published, containing 140 mutation-carriers, of whom 14 (10.0%) were asymptomatic (Supplementary Data 1; Figure S2). Thus, the rate of asymptomatic mutation carriers is significantly lower in Asian as compared to European cases (z = 5.28; p < 0.0001). We postulated that this difference could be accounted for by the allele frequency differences at the MSR1 CNV.

**Figure 2:**
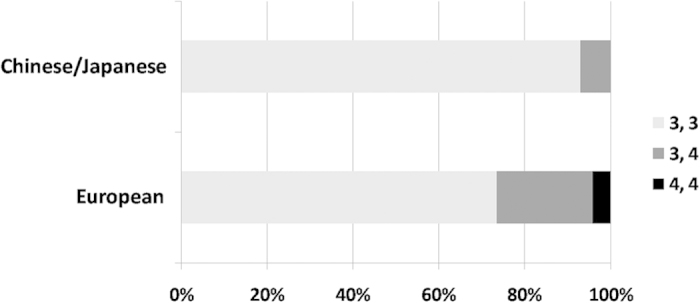
The frequency of *PRPF31* MSR1 cluster genotypes varies significantly between ethnic populations. In concordance with the differing rates of incomplete penetrance, the 4-copy allele was much rarer in Oriental populations compared to Caucasian populations.

Control samples from unrelated, unaffected individuals were tested for the MSR1 CNV at *PRPF31* (71 Asians and 283 Europeans). We demonstrated that only 5 Asians (7.0%) carried the 4-copy allele in the heterozygous state while no homozygotes were observed, corresponding to a minor allele frequency (MAF) of 0.035 (Fig. 2). In concordance with previously published data^17^, 22.2% of European controls were heterozygous (3/4) and 4.2% were homozygous (4/4), corresponding to a minor allele frequency (MAF) of 0.154 (Fig. 2). Thus, the frequency of the 4-copy allele is significantly lower in Asian than European individuals (z = 3.76; p < 0.0001). These results are consistent with the hypothesis that the 4-copy allele confers higher *PRPF31* gene expression and acts as the major protective factor. Therefore, in Asia, where asymptomatic mutation carriers are rare, the protective 4-copy allele is correspondingly rare. Although only a statistical correlation in the two populations studied, the principles of causation are maintained by this correlation. Taken as supporting evidence for the functional *in vitro* work, and the study in a large patient cohort, this finding strongly supports the conclusion that CNV of the MSR1 is the major *cis*-acting genetic element controlling incomplete penetrance in *PRPF31*-associated adRP.

### The MSR1 CNV is a generic modulator of basal promoter activity

**Figure 3:**
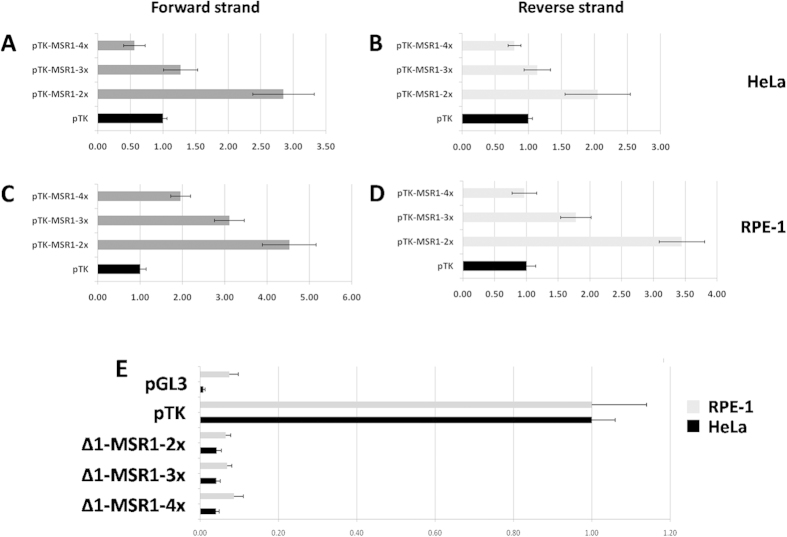
(**A–D**) Analysis of CNV of MSR1 in pTK promoter vector; the repeat cluster was cloned in forward strand orientation (**A,C**) and reverse strand orientation (**B,D**). This demonstrated a stepwise decrease in reporter activity with increasing MSR1 copy number. **(E)** Analysis of CNV of MSR1 in a pGL3-empty vector, demonstrating that the MSR1 elements have no intrinsic promoter activity. Data is presented as the mean ratio of the vector construct to pTK, a basic promoter vector, together with one standard deviation. The positive (pTK) and negative (pGL3) controls are also shown; x-axis – fold-induction over pTK; y-axis – vector.

To further assess the copy number effect of MSR1 on gene expression, we cloned DNA segments with 2, 3 or 4 copies of MSR1 (pTK-MSR1-2x, pTK-MSR1-3x and pTK-MSR1-4x respectively) upstream of the thymidine kinase (TK) minimal promoter in a pGL3 vector. In both cell lines assayed, increasing MSR1 copy number resulted in a significant decrease in luciferase reporter activity (Fig. 3A–D, Table 1). Importantly, this effect was independent of MSR1 element orientation. In the forward strand direction, pTK-MSR1-2x had 1.5 to 1.8-fold higher activity than pTK-MSR1-3x; the difference was significant in both cell lines tested (HeLa t = 8.94, p < 0.00001; RPE-1 t = 7.80 p < 0.00001). Also, pTK-MSR1-3x had 1.4 to 1.6 fold higher activity than pTK-MSR1-4x; significant in both cell lines tested (HeLa t = 7.39, p < 0.00001; RPE-1 t = 9.37, p < 0.00001). In the reverse strand direction, pTK-MSR1-2x had 1.9 to 2.2-fold higher activity than pTK-MSR1-3x, the difference being significant in both cell lines tested (HeLa t = 5.69, p < 0.00001; RPE-1 t = 13.36 p < 0.00001). Finally, pTK-MSR1-3x had 1.9 to 2.2-fold higher activity than pTK-MSR1-4x; this difference was significant in both cell lines tested (HeLa t = 4.75, p < 0.00008; RPE-1 t = 9.54, p < 0.00001). Subsequently, the MSR1 element was cloned into a pGL3-basic vector in 2-, 3- or 4-copies (Δ1-MSR1-2x, Δ1-MSR1-3x, Δ1-MSR1-4x, respectively). This analysis demonstrated that the isolated MSR1 cluster did not have intrinsic promoter activity, regardless of copy number (Fig. 3E, Table 1).

These experiments demonstrate that modulation of gene expression by MSR1 is not gene-specific, but it can control expression of any promoter element. In other words, the MSR1 element is a generic molecular switch that can control the expression of any gene in which it is contained. It is important to note that this effect is the reverse of the effect observed with the natural *PRPF31* promoter where increasing copy number increased reporter activity. This possibly suggests that the effect of the MSR1 cluster is dependent on the spatial relation of the repeats and the promoter, but not their orientation. It will be necessary to perform further reporter assays, with the MSR1 clusters at various genetic distances from the core promoter to fully explore this interesting hypothesis. In addition, we conclusively demonstrate that the MSR1 repeat is not a promoter element but a modulator of gene transcription.

### Genomic features of MSR1 elements

**Figure 4:**
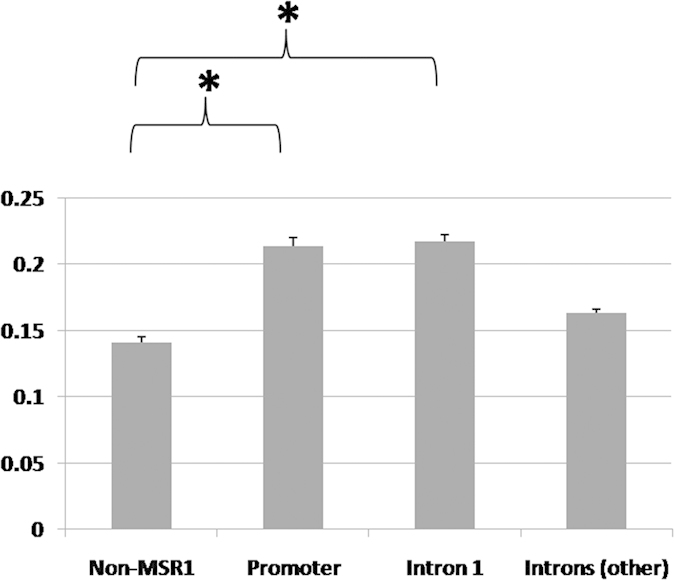
Analysis of population gene expression data demonstrated that genes containing an MSR1 cluster in the promoter or intron 1 had greater average variability than genes that did not contain an MSR1 cluster. Data is presented as the mean variability of each gene group (mean standard deviation) ± SEM; an asterisk indicates statistical significance.

Since MSR1 elements are widespread in the human genome (RepeatMasker browser; www.repeatmasker.org), we sought to examine how they might affect gene expression broadly. We examined population-level gene expression of four groups of chromosome 19 genes (i) genes containing an MSR1 cluster in the promoter, (ii) genes containing an MSR1 cluster in intron 1, (iii) genes containing an MSR1 cluster in other introns, and (iv) genes that did not contain an MSR1 cluster. Analysis of gene expression of the cognate group of genes in 270 control individuals, with published data from HapMap Phase II^19^, demonstrated that genes containing an MSR1 cluster in the promoter showed greater population-level variability, estimated as the mean standard deviation, than did genes without a MSR1 cluster (p < 0.04) (Fig. 4). Second, genes containing an MSR1 cluster in intron 1 also showed a significantly greater variability than non-MSR1 containing genes (p < 0.04) (Fig. 4). There was no observable difference between non-MSR1 containing genes and those with MSR1 elements in introns other than intron 1 (p = 0.23). These findings are consistent with the hypothesis that MSR1 elements regulate gene expression and that CNVs of MSR1 leads to the increased variation in gene expression. We believe that these variations represent an under-estimate of the true effect of MSR1 because it is unlikely that CNVs occurs in all MSR1 clusters in groups (i) and (ii).

**Figure 5:**
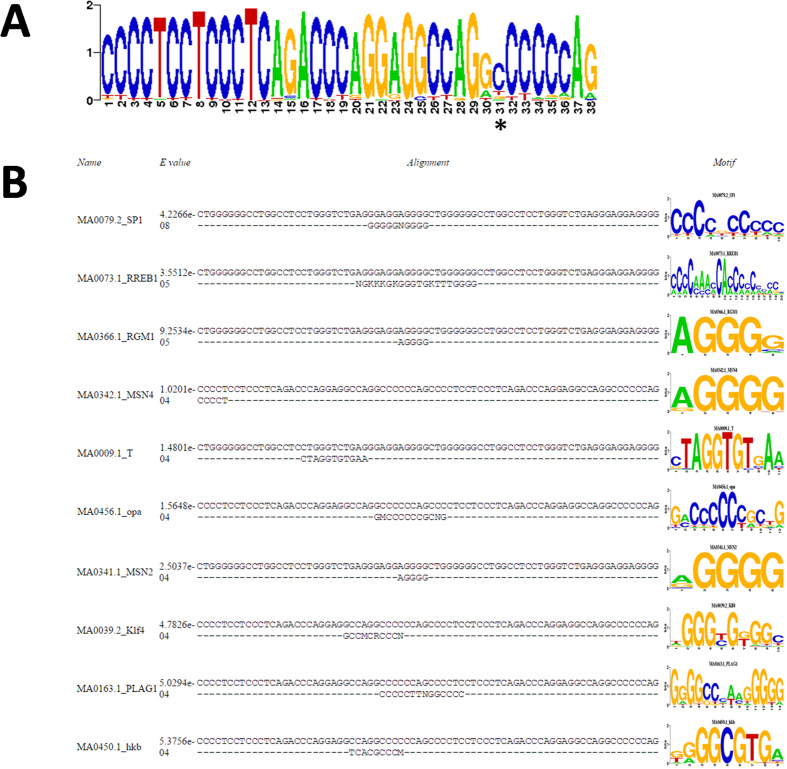
(**A**) Positional weight matrix of consensus sequence of MSR1 element, derived from 100 randomly selected MSR1 elements on chromosome 19. The asterisk indicates a C nucleotide that is frequently absent. (**B**) Prediction of TFBS using PWM of two consecutive MSR1 elements, allowing demonstration of junctional binding sites.

To understand how the MSR1 effect might be mediated, possibly through binding of a specific RNA or protein, we generated a consensus sequence positional weight matrix (PWM) using 100 randomly selected MSR1 elements from chromosome 19 (Fig. 5A). We used these sequences to identify potential transcription factor binding sites (TFBS), including at junctional binding sites when two or more MSR1 elements are present (Fig. 5B, Table 2). This demonstrated that there are a small number of putative TFBS within the MSR1 sequence, although only three potential sites for human transcription factors (SP1, RREB1, PLAG1). Further, the MSR1 sequence did not support the contention that it was a CpG island, so that epigenetic regulation by CpG methylation is unlikely to contribute to its gene expression effect.

## Discussion

The major finding from our study is that CNV in an MSR1 repeat element upstream of *PRPF31* modulates its transcriptional activity and thereby controls the phenotypic incomplete penetrance in *PRPF31*-associated adRP. This modulation, as a decrease in gene expression, occurs in all individuals that have at least one copy of the 3-repeat MSR1 allele. Although analysis of *PRPF31* gene expression in the retina has not been performed – and would be admittedly challenging – it is a specific prediction of our findings. In *PRPF31* mutation carriers there is one wildtype and one mutant allele; these mutant alleles are either loss-of-function or functional hypomorphs. Consequently, if such mutation bearers are homozygous for the 3-repeat MSR1 allele, *PRPF31* gene expression is reduced for both the mutant and wildtype allele, and these individuals can be symptomatic. In 3-repeat heterozygotes, gene expression will be reduced for the *PRPF31* allele in *cis* with the 3-repeat allele and so will have an impact on *PRPF31* gene expression either through the wildtype allele or further decrease for the *PRPF31* mutant. Our study confirms the theory that linked regulatory elements can modulate human disease penetrance^1^.

The primary benefit of this study is that our data provides an avenue for more accurate genetic counseling of families affected by *PRPF31*-associated adRP. The negative predictive value of a test assessing only the MSR1 copy number was estimated at up to 100%, meaning that if a *PRPF31* mutation carrier also carries a 4-copy allele in *cis* to the wildtype allele, we can say with very high certainty that they will not develop clinically relevant disease. Clearly, we need a replicate study before such quantitative statements can be clinically used. Nevertheless, the predictive accuracy of the test could be improved by integrating data relating to other SNPs that have been linked to the incomplete penetrance phenotype and/or expression QTLs (quantitative trait loci) for *PRPF31*.

Our investigations, however, have revealed a disparity between the dramatic effect observed *in vitro* (with up to 115-fold change in expression levels) and the modest effect seen *in vivo*^14^,^15^. Furthermore, a reasonable proportion of asymptomatic individuals carry two copies of the 3-copy allele and, in 3-copy allele homozygotes, *in vivo* expression cannot be completely abrogated, as homozygous mutation of *PRPF31* is embryonic lethal. Thus, additional genes and other factors must also play a role in the incomplete penetrance phenotype of *PRPF31*-associated adRP. These observations are in agreement with previous work suggesting polygenic control of gene expression and multiple modifying factors^15^,^16^. Other modifying factors might include negative regulation by CNOT3 and distant regulators on chromosome 14^16^,^20^. Finally, prior to nonsense-mediated decay of mutant *PRPF31* there is some increased expression of both *PRPF31* alleles in asymptomatic mutation carrying individuals^21^, suggesting that *trans*-acting factors might also control *PRPF31* expression.

What is the mechanism by which MSR1 elements exert their effect? Our work provides some initial clues. First, we demonstrate that the MSR1 element cluster is a generic modulator of basal promoter activity *without* possessing any intrinsic promoter activity. Second, like all classical enhancers, the effect of copy number is independent of orientation but dependent on the spatial relationship of the MSR1 cluster to the core promoter. Third, MSR1 elements throughout the genome that are close to the promoter or reside in intron 1 also affect gene expression. Consequently, by the classical definition, the MSR1 element acts as a modulator (enhancer/suppressor) rather than a repeat sequence that has been co-opted as a modulatory element.

Bioinformatics analyses show that the repeat element contains sequences that can putatively bind specific transcription factors including two sites generated when multiple MSR1 sequences are consecutively arranged in a cluster (junctional sites), so that CNVs of the elements might allow for differential binding and differential gene expression through TF-mediated enhancer activity. On the other hand, given that when cloned upstream to pTK basic promoter, variable copy number of the MSR1 repeat had the inverse effect, it seems more likely that mechanisms other than transcription factor binding might be involved. Since the element is not a site for CpG methylation, other epigenetic mechanisms might be considered. Given that MSR1 acts as an enhancer, we feel that it is very likely that DNA looping is critical to its effect on gene expression. This is supported by the dramatic effect of the spatial relation of the MSR1 cluster to the promoter. Further experiments to test these various hypotheses are necessary in order to fully understand how MSR1 modulates gene expression.

It is clear that MSR1 elements are likely to be an important genome-wide regulator of gene expression and it will be critical to establish which of the MSR1 clusters exhibit CNV in different human populations. The repeat element is a 36–38 bp minisatellite repeat that exists in highest density on chromosome 19q. The element appears to have arisen relatively recently in evolution, after the divergence of avian and mammalian lineages, and locus-specific conservation of elements has been reported^22^,^23^. It is crucial to examine whether CNV of elements underlies the population-level gene expression variability we observed at the large numbers of MSR1 containing loci across chromosome 19 and elsewhere. Examples of MSR1 CNV do exist: the kallikrein locus has two MSR1 clusters that have shown CNV, one within *KLK14* and one within *KLK4*^24^. Intriguingly, correlations between MSR1 copy number and breast and prostate adenocarcinoma were shown^24^. Given that aberrant kallikrein gene expression is key to the pathogenesis in many tumours, we predict that MSR1 CNVs might underlie increased susceptibility to cancer in these patients. In addition, hypermutation of MSR1 elements within cancer cells might further promote gene dysregulation. Finally, a cluster of MSR1 elements within the *TNNI3* promoter, conserved in both human and mouse has been reported^23^,^25^. Some authors have suggested selection pressure to maintain the elements as part of the functional *TNNI3* promoter since deletion of this degenerate cluster altered core *TNNI3* promoter activity in the mouse^25^,^26^.

These results allows us to hypothesize that variability of gene expression due to MSR1 CNV will be a modifier in many monogenic diseases and an important risk factor for the development of a multitude of polygenic disorders.

## Materials and Methods

### Amplification and cloning of BiP-MSR

The BiP-MSR fragment was amplified using template DNA from two individuals harbouring a 112 kb deletion at chromosome 19q13 and who were, therefore, hemizygous for the entire region of interest^18^. One individual (RP15011) was symptomatic, therefore representing a lower-expressing allele; this allele carried 3-copies of MSR1. The other individual (III.7) was asymptomatic, therefore representing a higher-expressing allele and this person carried 4-copies of MSR1. Blunt-ended PCR was performed using KOD polymerase (Novagen) and the following primer pair: GAAGAATCGTTTGGAACCCG and GATGTGGCCACCAAATAG (1398 bp, hg 19 co-ordinates chr19:54617996-54619393). pGL3-basic vector (Promega) was digested using SmaI restriction enzyme. The BiP-MSR insert was ligated into the digested vector and vectors of interest transformed into DH5-α cells; plasmids were isolated using standard Miniprep methods.

Dual-luciferase reporter assay was performed in HeLa and RPE-1 cell lines, as described in full previously^17^. Briefly, HeLa and RPE-1 cells were grown to 80–90% confluence and co-transfected with pGL3-MSR and pRenilla; each transfection was performed in quadruplicate. The cells were grown for 48 h, then lysed and dual-luciferase reporter assay was performed as instructed by the kit manufacturer (Promega). For each fragment, at least three separate transfections were performed. On each plate, a modified pGL3 plasmid containing basic TK promoter (pTK) was used as a positive control and pGL3-basic vector as a negative control. Data were disregarded if the pRenilla values for repeats varied more than  ± 10%.The data were firstly analyzed by calculating the ratio between pGL3-MSR and pRenilla; this calculation standardized for cell number and transfection efficiency. This ratio was then compared with the average value of the positive control (pTK), to generate the pGL3-MSR1:pTK ratio. Statistical significance was assessed using two-tailed Mann-Whitney U-test. The full dataset of luciferase assays are shown in Supplementary Data 2.

### Assessment of CNV in asymptomatic and symptomatic mutation carrying individuals

Genomic DNA was extracted from peripheral blood samples of *PRPF31* mutation-carrying individuals under standard conditions (72 individuals: 42 symptomatic and 29 asymptomatic). Informed consent had been obtained from all patients and asymptomatic relatives used in this study, and research was performed according to the tenets of the Declaration of Helsinki. The use of human DNA samples had been approved by the Moorfields Eye Hospital and UCL Institute of Ophthalmology Ethics Committee. PCR was performed using one labelled primer (5′FAM-GTTAGGGGTTTGGACTGC) and an unlabelled reverse primer (GATGTGGCCACCAAATAG). Labelled PCR product was sized using GeneScan 500 LIZ size standard (Invitrogen) on ABI3700 and data was analyzed on GeneMarker v1.7 (SoftGenetics LLC).

### Studies in different ethnic populations

All family trees affected by *PRPF31*-associated adRP with stated ethnicity that were published prior to January 2014 were assimilated, along with unpublished families from our laboratory. The asymptomatic and symptomatic mutation carriers were identified. Statistical significance was ascertained by comparison of two independent proportions (z-test).

CNV of MSR1 element was assayed as described above in genomic DNA of individuals of different ethnicity (Caucasian n = 283, Oriental n = 71). The DNA were from ECACC control panels of DNA, as well as samples collected from unrelated, healthy controls at Moorfields Eye Hospital, London, UK.

### Analysis in pTK and pGL3-basic vectors

To generate pTK-MSR1, pTK vector (Promega) was digested with SmaI restriction enzyme under standard conditions. PCR products (2- 3- or 4-copies of MSR1) were amplified from the BiP-MSR1 construct of individual III.7; these products were purified and the insert was ligated in both orientations into the digested vector and transformed as previously described. To generate Δ1-MSR1 constructs, pGL3-basic vector (Promega) underwent the same process as that of pTK-MSR1. Luciferase assay was performed in RPE-1 and HeLa cell lines, as previously described for BiP-MSR.

### Analysis of population-level gene expression variability

The expression of chromosome 19 genes containing MSR1 elements in (i) promoter (ii) intron 1 or (iii) other introns was compared to a random sample of chromosome 19 genes that did not contain MSR1 elements (chosen from an alphabetical list using a random number generator). Gene expression data from the 270 individuals of various ethnicities from the HapMap phase II project was downloaded from the Gene Expression Omnibus (GEO) database (http://www.ncbi.nlm.nih.gov/geo)^19^. The mean and standard deviation of each gene of interest was calculated. The mean standard deviation (e.g. variability) and standard error of the mean (SEM) were then calculated for the four study groups (promoter, intron 1, other introns, non-MSR1). Statistical significance was assessed using Student’s T-test for two independent means.

### Bioinformatic analysis of TFBSs and epigenetic regulation

The sequence of 100 randomly selected MSR1 elements was obtained and the frequency of nucleotides at each position was recorded to generate a consensus positional weight matrix. TFBSs were identified using a consecutive series of two consensus MSR1 PWMs in the STAMP database on routine settings (available at http://www.benoslab.pitt.edu/stamp)^27^. CpG island analysis was performed using the EMBOSS Newcpgreport tool (available at http://www.ebi.ac.uk/Tools/seqstats/emboss_newcpgreport/)^28^.

## Additional Information

**How to cite this article**: Rose, A. M. *et al.* Transcriptional regulation of *PRPF31* gene expression by MSR1 repeat elements causes incomplete penetrance in retinitis pigmentosa. *Sci. Rep.* **6**, 19450; doi: 10.1038/srep19450 (2016).

## Supplementary Material

Supplementary Information

## Figures and Tables

**Table 1:** Luciferase assay results of tested fragments in two cell lines, presented as the mean ratio of the constructs to pTK (a basic promoter vector), together with one standard deviation.

	Fold induction over pTK	
	HeLa	RPE-1
BiP-MSR-3x	0.20 ± 0.07	0.07 ± 0.03
BiP-MSR-4x	10.63 ± 1.63	8.05 ± 1.36
pTK-MSR-2x (+)	2.85 ± 0.49	4.53 ± 0.64
pTK-MSR-3x (+)	1.27 ± 0.26	3.11 ± 0.35
pTK-MSR-4x (+)	0.56 ± 0.16	1.96 ± 0.24
pTK-MSR-2x (−)	2.05 ± 0.49	3.45 ± 0.36
pTK-MSR-3x (−)	1.14 ± 0.20	1.78 ± 0.24
pTK-MSR-4x (−)	0.79 ± 0.10	0.97 ± 0.19
Δ1-MSR-2x	0.042 ± 0.012	0.065 ± 0.013
Δ1-MSR-3x	0.041 ± 0.010	0.069 ± 0.012
Δ1-MSR-4x	0.040 ± 0.009	0.086 ± 0.024
pTK (positive control)	1.00 ± 0.06	1.00 ± 0.14
pGL3 (negative control)	0.008 ± 0.004	0.074 ± 0.023

**Table 2:**
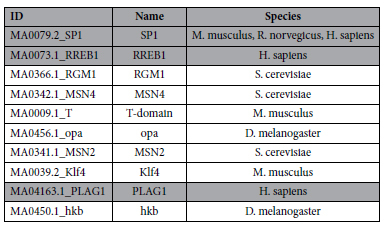
Bioinformatic prediction of TFBSs based on consensus PWM of two consecutive MSR1 elements.

The three factors highlighted in grey represent human transcription factors.

## References

[b1] Chakravarti A. & Kapoor A. Genetics- Mendelian puzzles. Science. 335, 930–931 (2012). 22362999 10.1126/science.1219301

[b2] Al-Maghtheh M. *et al.* Evidence for a major retinitis pigmentosa locus on 19q13.4 (RP11) and association with a unique bimodal expressivity phenotype. Am J Hum Genet. 59, 864–871 (1996). 8808602 PMC1914817

[b3] Vithana E. N. *et al.* A human homolog of yeast pre-mRNA splicing gene, *PRP31*, underlies autosomal dominant retinitis pigmentosa on chromosome 19q13.4 (RP11). Mol Cell. 8, 375–381 (2001). 11545739 10.1016/s1097-2765(01)00305-7

[b4] Weidenhammer E. M., Ruiz-Noriega M. & Woolford J. L. Jr. Prp31p promotes the association of the U4/U6 × U5 tri-snRNP with prespliceosomes to form spliceosomes in Saccharomyces cerevisiae. Mol Cell Biol. 17, 3580–3588 (1997). 9199293 10.1128/mcb.17.7.3580 PMC232211

[b5] Makarova O. V., Makarov E. M., Liu S., Vornlocher H. P. & Lührmann R. Protein 61K, encoded by a gene (*PRPF31*) linked to autosomal dominant retinitis pigmentosa, is required for U4/U6*U5 tri-snRNP formation and pre-mRNA splicing. EMBO J. 21, 1148–1157 (2002). 11867543 10.1093/emboj/21.5.1148 PMC125353

[b6] Liu S., Rauhut R., Vornlocher H. P. & Lührmann R. The network of protein-protein interactions within the human U4/U6.U5 tri-snRNP. RNA. 12, 1418–1430 (2006). 16723661 10.1261/rna.55406 PMC1484429

[b7] Liu S. *et al.* Binding of the human Prp31 Nop domain to a composite RNA-protein platform in U4 snRNP. Science. 316, 115–120 (2007). 17412961 10.1126/science.1137924

[b8] Waseem N. H., al. Mutations in the gene coding for the pre-mRNA splicing factor, *PRPF31*, in patients with autosomal dominant retinitis pigmentosa. Invest. Ophthalmol. Vis. Sci. 48, 1330–1334 (2007). 17325180 10.1167/iovs.06-0963

[b9] Vithana E., Al-Maghtheh M., Bhattacharya S. S. & Inglehearn C. F. RP11 is the second most common locus for dominant retinitis pigmentosa. J. Med. Genet. 35, 174–175 (1998). 9556378 10.1136/jmg.35.2.174-a PMC1051233

[b10] Audo I. *et al.* Prevalence and novelty of *PRPF31* mutations in French autosomal dominant rod-cone dystrophy patients and a review of published reports. BMC Med. Genet. 11, 145 (2010). 20939871 10.1186/1471-2350-11-145 PMC2984399

[b11] Xu F. *et al.* Novel *PRPF31* mutations associated with Chinese autosomal dominant retinitis pigmentosa patients. Mol. Vis. 18, 3021 (2012). 23288994 PMC3534138

[b12] McGee T. L., Devoto M., Ott J., Berson E. L. & Dryja T. P. Evidence that the penetrance of mutations at the RP11 locus causing dominant retinitis pigmentosa is influenced by a gene linked to the homologous RP11 allele. Am. J. Hum. Genet. 61, 1059–1066 (1997). 9345108 10.1086/301614 PMC1716046

[b13] Rose A. M. *et al.* Dominant *PRPF31* mutations are hypostatic to a recessive CNOT3 polymorphism in retinitis pigmentosa: a novel phenomenon of “linked trans-acting epistasis”. Ann. Hum. Genet. 78, 62–71 (2014). 24116917 10.1111/ahg.12042 PMC4240469

[b14] Vithana E. N. *et al.* Expression of *PRPF31* mRNA in patients with autosomal dominant retinitis pigmentosa: a molecular clue for incomplete penetrance? Invest. Ophthalmol. Vis. Sci. 44, 4204–4209 (2003). 14507862 10.1167/iovs.03-0253

[b15] Rivolta C. *et al.* Variation in retinitis pigmentosa-11 (*PRPF31* or RP11) gene expression between symptomatic and asymptomatic patients with dominant RP11 mutations. Hum. Mutat. 27, 644–653 (2006). 16708387 10.1002/humu.20325

[b16] Rio Frio T., Civic N., Ransijn A., Beckmann J. S. & Rivolta C. Two trans-acting eQTLs modulate the penetrance of *PRPF31* mutations. Hum. Mol. Genet. 17, 3154–3165 (2008). 18640990 10.1093/hmg/ddn212

[b17] Rose A. M. *et al.* Expression of *PRPF31* and TFPT: regulation in health and retinal disease. Hum. Mol. Genet. 21, 4126–4137 (2012). 22723017 10.1093/hmg/dds242

[b18] Rose A. M., Mukhopadhyay R., Webster A. R., Bhattacharya S. S.& Waseem N. H. A 112 kb deletion in chromosome 19q13.42 leads to retinitis pigmentosa. Invest. Ophthalmol. Vis. Sci. 52, 6597–6603 (2011). 21715351 10.1167/iovs.11-7861

[b19] International HapMap Consortium *et al.* A second generation human haplotype map of over 3.1 million SNPs. Nature. 449, 851–61. (2007). 17943122 10.1038/nature06258 PMC2689609

[b20] Venturini G., Rose A. M., Shah A. Z., Bhattacharya S. S. & Rivolta C. CNOT3 is a modifier of *PRPF31* mutations in retinitis pigmentosa with incomplete penetrance. PLoS Genet. 8, e1003040 (2012). 23144630 10.1371/journal.pgen.1003040 PMC3493449

[b21] Rio Frio T. *et al.* Premature termination codons in *PRPF31* cause retinitis pigmentosa via haploinsufficiency due to nonsense-mediated mRNA decay. J Clin Invest. 118, 1519–31 (2008). 18317597 10.1172/JCI34211 PMC2262031

[b22] Das H. K., Jackson C. L., Miller D. A., Leff T. & Breslow J. L. The human apolipoprotein C-II gene sequence contains a novel chromosome 19-specific minisatellite in its third intron. J Biol Chem. 262, 4787–93 (1987). 3558370

[b23] Bhavsar P. K., Brand N. J., Yacoub M. H. & Barton P. J. Isolation and characterization of the human cardiac troponin I gene (TNNI3). Genomics. 35, 11–23 (1996). 8661099 10.1006/geno.1996.0317

[b24] Yousef G. M., Bharaj B. S., Yu H., Poulopoulos J. & Diamandis E. P. Sequence analysis of the human kallikrein gene locus identifies a unique polymorphic minisatellite element. Biochem. Biophys. Res. Commun. 285, 1321–1329 (2001). 11478802 10.1006/bbrc.2001.5321

[b25] Cullen M. E., Dellow K. A. & Barton P. J. Structure and regulation of human troponin genes. Mol. Cell. Biochem. 263, 81–90 (2004). 27520667 10.1023/B:MCBI.0000041850.37415.b8

[b26] Di Lisi R. *et al.* Combinatorial cis-acting elements control tissue-specific activation of the cardiac troponin I gene *in vitro* and *in vivo*. J. Biol. Chem. 273, 25371–25380 (1998). 9738004 10.1074/jbc.273.39.25371

[b27] Mahony S.& Benos P. V. STAMP: a web tool for exploring DNA-binding motif similarities. Nucleic Acids Res. 35, W253–8 (2007). 17478497 10.1093/nar/gkm272 PMC1933206

[b28] McWilliam H. *et al.* Analysis Tool Web Services from the EMBL-EBI. Nucleic Acids Res. 41, W597–600 (2013). 23671338 10.1093/nar/gkt376 PMC3692137

