# Supplementary material for: Transcriptional regulation of PRPF31 gene expression by MSR1 repeat elements causes incomplete penetrance in retinitis pigmentosa
Source: Sci Rep. 2016 Jan 19;6:19450. doi: 10.1038/srep19450 (PMC4725990; doi:10.1038/srep19450)
Supplement: Supplementary Information [file srep19450-s1.pdf]

SUPPLEMENTARY DATA

Transcriptional regulation of *PRPF31* gene expression by MSR1 repeat elements causes incomplete penetrance in retinitis pigmentosa

Anna M. Rose, Amna Z. Shah, Guilia Venturini, Abhay Krishna, Aravinda Chakravarti, Carlo Rivolta, Shomi S. Bhattacharya

Supplementary Figure 1:
European *PRPF31*-mutation carrying families. Symptomatic mutation carriers are shown in black, whereas asymptomatic mutation carriers are indicated by a black dot.

A. RP3835, 112kb deletion, Rose et al. Invest Ophthalmol Vis Sci. 52:6597-603.
B. S240, c.732-737 delins 20bp, Martinez-Gimeno et al. Invest Ophthalmol Vis Sci. 44:2171-7.
C. M637, c.769-770 insA, Martinez-Gimeno et al. Invest Ophthalmol Vis Sci. 44:2171-7.
D. M368, c.828-829 delCA, Martinez-Gimeno et al. Invest Ophthalmol Vis Sci. 44:2171-7.
E. Patient 85, c.527+3 A>G, Waseem et al. Invest Ophthalmol Vis Sci. 48:1330-4.
F. Patient 2076, p.Glu27X, Waseem et al. Invest Ophthalmol Vis Sci. 48:1330-4.
G. Patient 15590, c.527+3 A>G, Waseem et al. Invest Ophthalmol Vis Sci. 48:1330-4.
H. Patient 16220, c.1146+2 T>C, Waseem et al. Invest Ophthalmol Vis Sci. 48:1330-4.
I. Patient 16722, c.528-1 G>A, Waseem et al. Invest Ophthalmol Vis Sci. 48:1330-4.
J. Patient 2181, p.Thr138Lys, Waseem et al. Invest Ophthalmol Vis Sci. 48:1330-4.
K. F273, c.269_273 del, Audo et al. BMC Med Genet. 11:145.
L. Swedish, 59kb deletion, Kohn et al. Eur J Hum Genet. 17:651-5.
M. F1706, c.709_734dup, Audo et al. BMC Med Genet. 11:145.
N. American, 1374+654C>G, Rio Frio et al. Hum Mutat. 30(9):1340-7.
O. F405, c. 527+2 T>C, Audo et al. BMC Med Genet. 11:145.
P. F28, c.666dup, Audo et al. BMC Med Genet. 11:145.
Q. F700, c.873_897dup, Audo et al. BMC Med Genet. 11:145.
R. F108, c.997delC, Audo et al. BMC Med Genet. 11:145.
S. RP1907, c.527+3 A>G, Waseem et al. Waseem et al. Invest Ophthalmol Vis Sci. 48:1330-4.
T. RP677, IVS6-3 to-45 del, Waseem et al. Waseem et al. Invest Ophthalmol Vis Sci. 48:1330-4.
U. Greek, p.R211Q, unpublished
V. AD29, c.C646G, Vithana et al. Mol Cell. 8:375-81.
W. Canadian, DelC at transcription start site, Rose et al. Hum Mol Genet. 21:4126-37.
X. Romani, IVS6+1 G>T, Chakarova et al. Mol Vis. 12:909-14.
Y. AD24, c.527+3 A>G, unpublished
Z. AD5, 11bp deletion, Vithana et al. Mol Cell. 8:375-81.
AA. AD2, 11kb deletion, Rose et al. Invest Ophthalmol Vis Sci. 52:6597-603.
AB. AD11, IVS1+54 T>C, unpublished

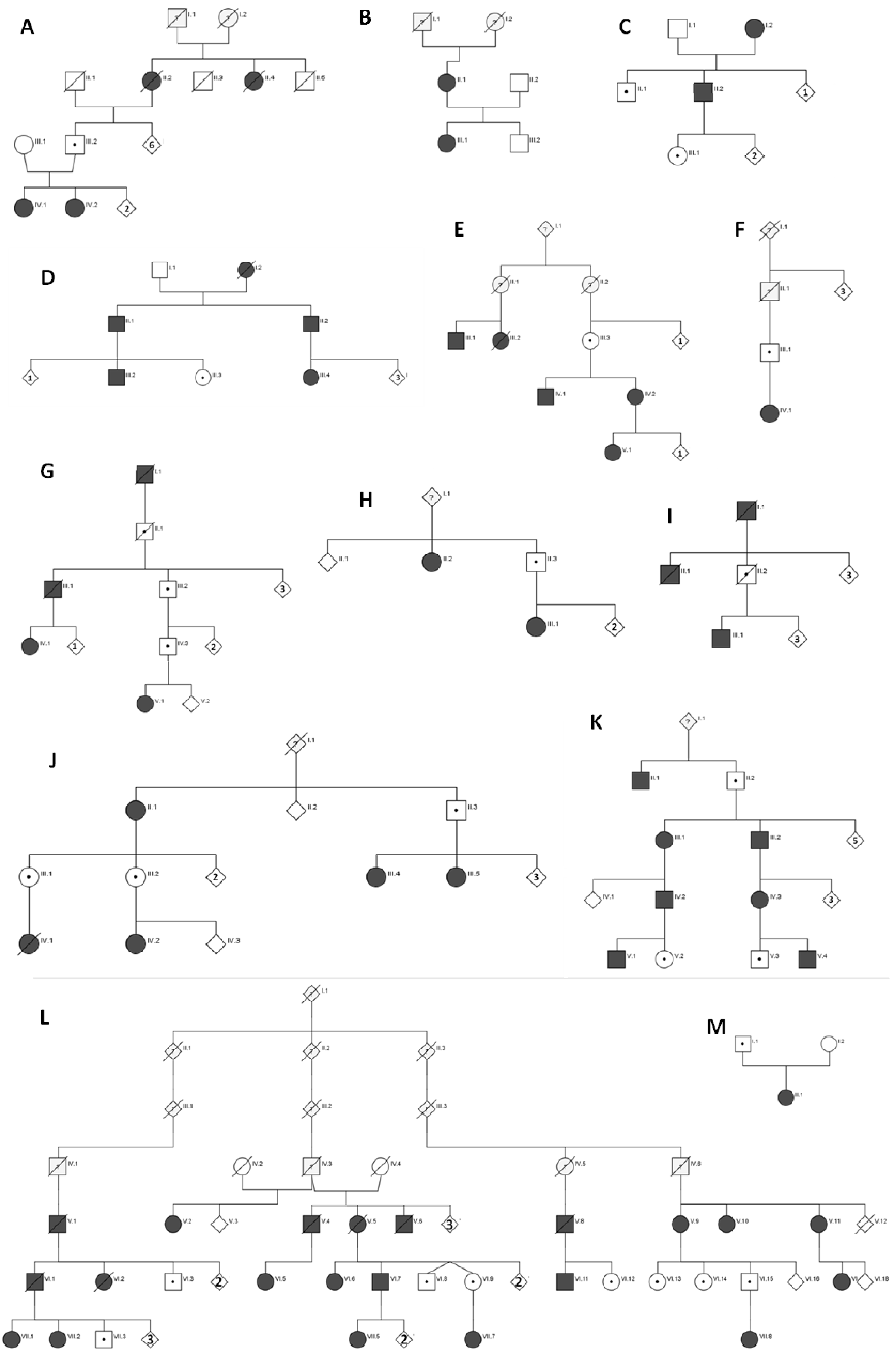
A
B
C
D
E
F
G
H
I
J
K
L
M

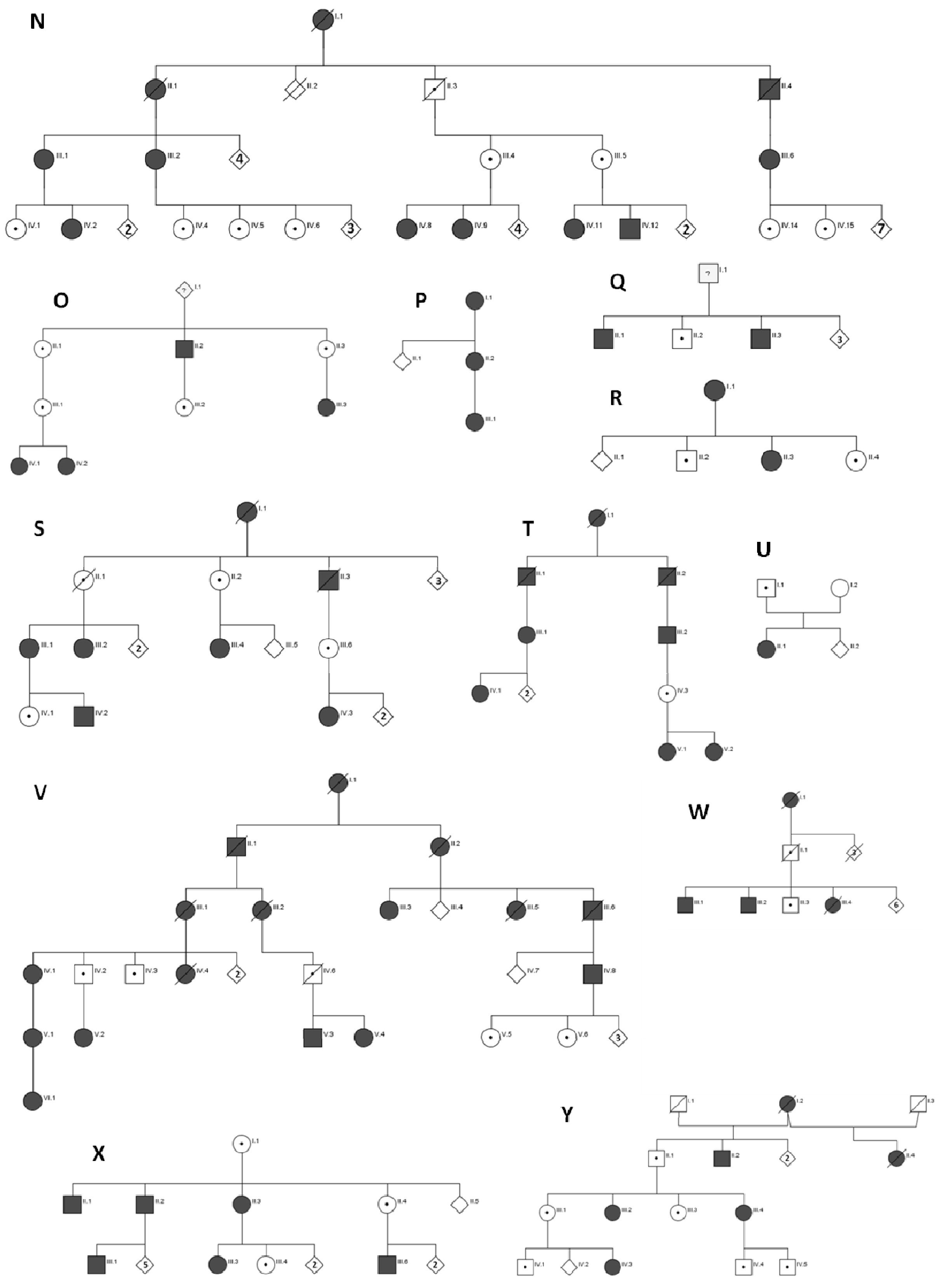
N
O
P
Q
R
S
T
U
V
W
X
Y

Z

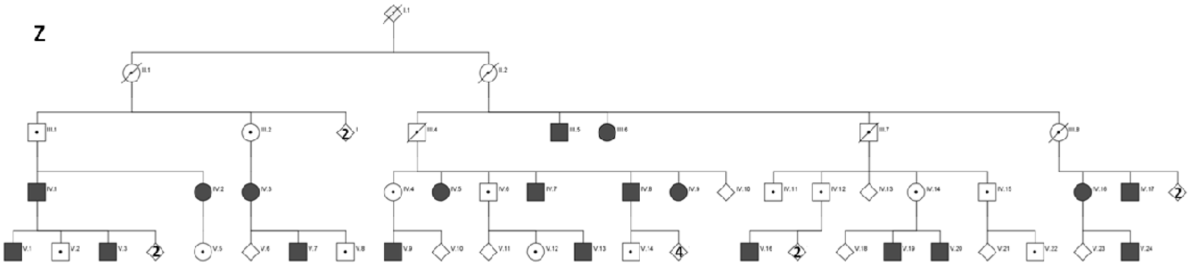

AA

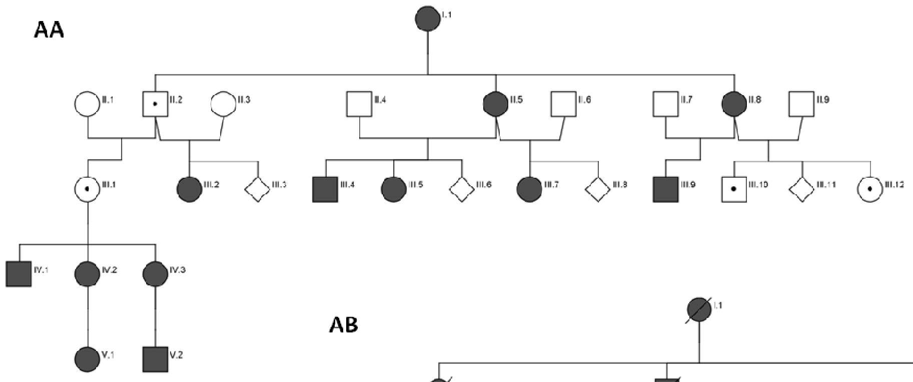

AB

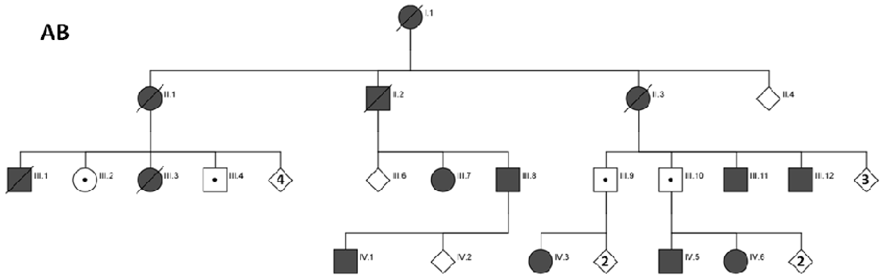

Supplementary Figure 2
East Asian (Japanese/Chinese) *PRPF31*-mutation carrying families. Symptomatic mutation carriers are shown in black, whereas asymptomatic mutation carriers are indicated by a black dot.

A. Chinese, c. delAA, Yang et al. BMJ Open. 3:e004030.
B. ADRP-HT, c.196_197delAA, Xu et al. Mol Vis. 18:3021
C. ADRP-LG, c.544_618del75bp, Xu et al. Mol Vis. 18:3021
D. ADRP-XL, c.T895C, Xu et al. Mol Vis. 18:3021
E. ADRP-LLN, c.615delC, Xu et al. Mol Vis. 18:3021
F. Chinese, 12bp deletion, Wang et al. Am J Med Genet A. 121A:235-9.
G. Family 1, 1142delG, Sato et al. Am J Ophthalmol. 140:537-40.
H. Family 2, 1155-1159indel, Sato et al. Am J Ophthalmol. 140:537-40.
I. Chinese, IVS5-1G>A, Xia et al. Mol Vis. 10:361-5.
J. Family 3, IVS6-3 to -45 del, Sato et al. Am J Ophthalmol. 140:537-40.
K. Japanese, c.1142delG, Taira et al. Jpn J Ophthalmol.51:45-8
L. Chinese, IVS1+1G>T, Liu et al. Biochem Biophys Res Commun. 367:420-6.

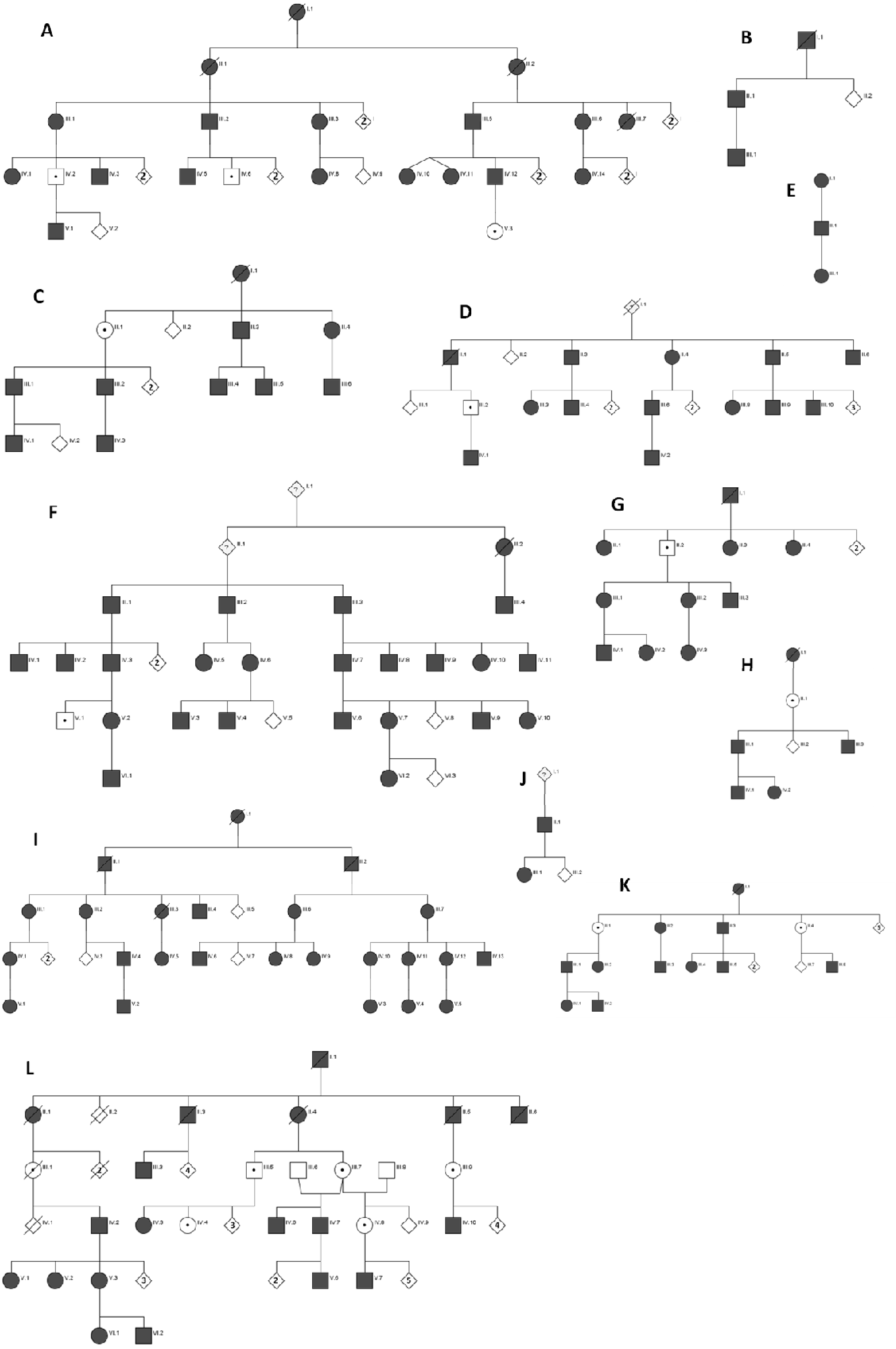
A
B
E
C
D
F
G
H
J
I
K
L

Fragment	Cell Line	Firefly	Renilla	Ratio	Ratio (adjusted)	Ratio:pTK
pGL3-basic	HeLa	14	117624	0.000119023	0.119023329	0.002767984
(negative control)		12	91131	0.000131679	0.131678573	0.003062292
		21	107741	0.000194912	0.194911872	0.004532834
		29	140123	0.000206961	0.206961027	0.004813047
		33	121555	0.000271482	0.271482045	0.006313536
		38	122809	0.000309424	0.309423576	0.007195897
		48	133476	0.000359615	0.359615212	0.008363144
		59	120672	0.000488929	0.488928666	0.011370434
		70	127254	0.000550081	0.55008094	0.01279258
		79	123933	0.000637441	0.637441198	0.014824214
		84	129654	0.000647878	0.647878199	0.015066935
pTK	HeLa	1553	36505	0.042542118	42.54211752	0.98935157
(positive control)		2826	61386	0.046036556	46.03655557	1.070617571
		2825	68241	0.0413974	41.39740039	0.962730242
		3379	72188	0.046808334	46.8083338	1.088565902
		3154	63474	0.049689637	49.6896367	1.155572947
		2018	44001	0.045862594	45.86259403	1.066571954
		2433	53834	0.045194487	45.19448676	1.051034576
		1758	36688.4	0.047917053	47.9170528	1.114350065
		1512	35467.6	0.042630457	42.63045709	0.991405979
		1639	39659.6	0.041326742	41.32674223	0.961087029
pGL3-basic	RPE-1	66	135495	0.000487103	0.487102845	0.036350959
(negative control)		70	124005	0.000564493	0.564493367	0.042126371
		86	134868	0.000637661	0.637660527	0.047586607
		71	79219	0.00089625	0.896249637	0.066884301
		58	72427	0.000800806	0.800806329	0.059761666
		88	77754	0.001131775	1.131774571	0.084460789
		69	53898	0.001280196	1.280195926	0.095537009
		53	75363	0.000703263	0.703262874	0.052482304
		81	71463	0.001133454	1.133453675	0.084586095
		83	82464	0.0010065	1.006499806	0.075111926
		88	73612	0.001195457	1.195457262	0.089213229
		74	95947	0.000771259	0.771259133	0.057556652
		137	97145	0.001410263	1.410263009	0.105243508
		130	97491	0.001333456	1.333456422	0.099511673
		111	89996	0.001233388	1.233388151	0.092043892
pTK	RPE-1	611	57982	0.010537753	10.5377531	0.786399485
(positive control)		863	73764	0.011699474	11.699474	0.873095074
		145	12346	0.011744695	11.74469464	0.876469749
		99	8325	0.011891892	11.89189189	0.887454619
		454	37162	0.012216781	12.21678058	0.911700043
		299	22923	0.013043668	13.04366793	0.973408055
		465	32411	0.014346981	14.34698096	1.070670221
		345	23357	0.014770733	14.77073254	1.102293473
		461	29724	0.015509353	15.50935271	1.157414381
		27	1708	0.015807963	15.80796253	1.179698696
		502	31494	0.015939544	15.93954404	1.189518212

Individual	Fragment	Cell Line	Firefly	Renilla	Ratio	Adjusted ratio	Ratio:pTK
RP15011	BiP-MSR-3x	RPE-1	24	49509	0.00048476	0.484760347	0.036176145
			26	49690	0.000523244	0.523244114	0.039048068
			25	45570	0.000548607	0.548606539	0.040940787
			4	7193	0.000556096	0.556096205	0.041499717
			43	65187	0.000659641	0.659640726	0.04922692
			33	49006	0.000673387	0.673386932	0.050252756
			41	48051	0.00085326	0.853260078	0.063676125
			42	48532	0.000865408	0.86540839	0.064582716
			55	61759	0.000890558	0.890558461	0.066459587
			66	72606	0.000909016	0.909015784	0.067836999
			38	40175	0.000945862	0.945861854	0.070586706
			61	61803	0.000987007	0.987007103	0.073657247
			48	48429	0.000991142	0.991141671	0.073965796
			47	44649	0.001052655	1.052655155	0.078556355
			52	49191	0.001057104	1.057103942	0.078888354
			75	68462	0.001095498	1.095498233	0.081753599
			79	62395	0.001266127	1.266127094	0.094487097
			5	2402	0.002081599	2.081598668	0.155343184
III.7	BiP-MSR1-4x	RPE-1	72	892	0.080717489	80.71748879	6.023693193
			914	11250	0.081248055	81.24805547	6.063287722
			1338	15946	0.083910821	83.91082123	6.262001585
			1497	17623	0.084945809	84.94580945	6.339239511
			1164	11492	0.101287852	101.2878524	7.558794957
			2419	23786	0.101698478	101.6984781	7.589438664
			1172	11427	0.102568591	102.5685906	7.65437243
			1358	12970	0.104707198	104.7071977	7.813969974
			2015	18896	0.106639147	106.6391469	7.95814529
			2006	18589	0.107913282	107.913282	8.053230004
			930	7923	0.117387188	117.3871884	8.760237939
			1567	13159	0.119081997	119.0819971	8.886716202
			125	1035	0.120772947	120.7729469	9.012906482
			218	1777	0.122678672	122.6786719	9.15512477
			1929	15596	0.12368556	123.6855604	9.230265702
			1575	11647	0.135227956	135.2279557	10.09163848
			802	5738	0.139769955	139.7699547	10.43059363
RP15011	BiP-MSR1-3x	HeLa	10	1985	0.005037783	5.037783375	0.117157753
			13	2133	0.006094702	6.094702297	0.141737263
			11	1744	0.006307339	6.30733945	0.146682313
			14	2020	0.006930693	6.930693069	0.161178909
			16	2200	0.007272727	7.272727273	0.169133192
			11	1391	0.00790798	7.907979871	0.183906509
			12	1424	0.008426966	8.426966292	0.19597596
			19	2076	0.009152216	9.1522158	0.212842228
			10	979	0.010214505	10.2145046	0.237546619
			15	12538	0.001196363	11.96363056	0.278223967
			13	951	0.013669821	13.66982124	0.31790282
			19	1371	0.013858497	13.85849745	0.322290638
III.7	BiP-MSR1-4x	HeLa	342	1020	0.335359874	335.3598745	7.799066848
			368	963	0.381980486	381.9804858	8.883267111

			507	1182	0.42893401	428.9340102	9.975209538
			577	1345	0.428996283	428.9962825	9.976657733
			532	1228	0.433083686	433.0836861	10.07171363
			455	996	0.45691906	456.9190601	10.62602465
			523	1140	0.45877193	458.7719298	10.66911465
			717	1453	0.493393889	493.3938893	11.4742765
			570	1138	0.500966778	500.966778	11.65039019
			502	1001	0.501498501	501.4985015	11.66275585
			601	1088	0.552389706	552.3897059	12.84627223
			646	1139	0.567064607	567.0646067	13.18754899
			718	1258.8	0.570384493	570.3844932	13.26475566
			725	1269	0.571315997	571.3159968	13.28641853

Fragment	Orientation	Firefly	Renilla	Ratio	Ratio:pTK
pTK-MSR1-2x	(+)	50	532	93.98496241	2.09320629
pTK-MSR1-2x	(+)	87	800	108.75	2.422048998
pTK-MSR1-2x	(+)	75	671	111.7734724	2.489386914
pTK-MSR1-2x	(+)	181	1580	114.556962	2.551380001
pTK-MSR1-2x	(+)	161	1340	120.1492537	2.675929927
pTK-MSR1-2x	(+)	176	1393	126.3460158	2.813942445
pTK-MSR1-2x	(+)	182	1411	128.9865344	2.872751322
pTK-MSR1-2x	(+)	176	1308	134.5565749	2.996805678
pTK-MSR1-2x	(+)	238	1571	151.4958625	3.374072662
pTK-MSR1-2x	(+)	88	575	153.0434783	3.408540719
pTK-MSR1-2x	(+)	213	1305	163.2183908	3.63515347
pTK-MSR1-2x	(-)	159	1512	105.1587302	2.34206526
pTK-MSR1-2x	(-)	47	727	64.64924347	1.43984952
pTK-MSR1-2x	(-)	52	800	65	1.44766147
pTK-MSR1-2x	(-)	115	1607	71.56191661	1.593806606
pTK-MSR1-2x	(-)	37	498.667	74.19786096	1.652513607
pTK-MSR1-2x	(-)	47	617.667	76.09282245	1.694717649
pTK-MSR1-2x	(-)	67	691	96.96092619	2.159486107
pTK-MSR1-2x	(-)	99	928.7	106.6006245	2.37417872
pTK-MSR1-2x	(-)	193	1747	110.4751002	2.460469937
pTK-MSR1-2x	(-)	96	809	118.6650185	2.642873464
pTK-MSR1-2x	(-)	219	1780	123.0337079	2.740171667
pTK-MSR1-3x	(+)	93	2275	0.040873731	0.910328083
pTK-MSR1-3x	(+)	44	1036	0.042471042	0.94590295
pTK-MSR1-3x	(+)	82	1845	0.044434811	0.989639441
pTK-MSR1-3x	(+)	64	1117	0.057296329	1.276087516
pTK-MSR1-3x	(+)	69	1123	0.061442565	1.368431282
pTK-MSR1-3x	(+)	17	275	0.061818182	1.376796922
pTK-MSR1-3x	(+)	71	1134	0.062610229	1.394437178
pTK-MSR1-3x	(+)	25	359	0.069637883	1.550955078
pTK-MSR1-3x	(+)	76	1049	0.072449952	1.613584685
pTK-MSR1-3x	(-)	87	2147	0.040521658	0.902486818
pTK-MSR1-3x	(-)	65	1604	0.040523691	0.902532088
pTK-MSR1-3x	(-)	75	1772	0.042325056	0.942651591
pTK-MSR1-3x	(-)	113	2418	0.046732837	1.040820424
pTK-MSR1-3x	(-)	82	1666.5	0.04920492	1.095877962

pTK-MSR1-3x	(-)	43	859	0.050058207	1.114882121
pTK-MSR1-3x	(-)	31	605	0.051239669	1.14119531
pTK-MSR1-3x	(-)	82	1538	0.053315995	1.187438637
pTK-MSR1-3x	(-)	101	1795	0.056267409	1.253171703
pTK-MSR1-3x	(-)	42	649	0.064714946	1.44131283
pTK-MSR1-3x	(-)	168	2495.6	0.067318481	1.499298007
pTK-MSR1-4x	(+)	30	1918	0.015641293	0.348358419
pTK-MSR1-4x	(+)	11	662	0.016616314	0.370073813
pTK-MSR1-4x	(+)	33	1749	0.018867925	0.420221036
pTK-MSR1-4x	(+)	14	651	0.021505376	0.478961611
pTK-MSR1-4x	(+)	47	2149	0.021870638	0.487096604
pTK-MSR1-4x	(+)	32	1411	0.022678951	0.505099134
pTK-MSR1-4x	(+)	125	4491	0.027833445	0.619898545
pTK-MSR1-4x	(+)	62	2157.7	0.0287343	0.639962147
pTK-MSR1-4x	(+)	20	635	0.031496063	0.701471336
pTK-MSR1-4x	(+)	202	5805.5	0.034794591	0.774935219
pTK-MSR1-4x	(+)	75	1988.8	0.037711183	0.839892709
pTK-MSR1-4x	(-)	72	2370	0.030379747	0.676609061
pTK-MSR1-4x	(-)	56	1772	0.031602709	0.703846521
pTK-MSR1-4x	(-)	61	1899	0.03212217	0.715415803
pTK-MSR1-4x	(-)	43	1325	0.03245283	0.722780182
pTK-MSR1-4x	(-)	26	758	0.034300792	0.763937451
pTK-MSR1-4x	(-)	35	934.4	0.037457192	0.834235897
pTK-MSR1-4x	(-)	70	1813	0.038610039	0.859911773
pTK-MSR1-4x	(-)	79	1969	0.040121889	0.89358328
pTK-MSR1-4x	(-)	73	1706	0.042790152	0.953010076
